# Variations on a theme: crystal forms of the amino-acid transporter MhsT

**DOI:** 10.1107/S2053230X22007154

**Published:** 2022-07-26

**Authors:** Caroline Neumann, Dorota Focht, Sofia Trampari, Joseph A. Lyons, Poul Nissen

**Affiliations:** a Center for Structural Biology, Department of Molecular Biology and Genetics, Aarhus University, Gustav Wieds Vej 10C, Aarhus C, DK-8000 Aarhus, Denmark; b DANDRITE, Nordic EMBL Partnership for Molecular Medicine, Denmark; Baylor College of Medicine, Houston, USA

**Keywords:** pseudomerohedral twinning, pseudosymmetry, translational NCS, X-ray crystallography, translational symmetry, membrane proteins, bacterial amino-acid transporters, MhsT

## Abstract

The bacterial amino-acid transporter MhsT from the SLC6A family has been crystallized in complex with different substrates in order to understand the determinants of the substrate specificity of the transporter. Surprisingly, crystals of the different MhsT–substrate complexes showed interrelated but different crystal-packing arrangements. Space-group assignment and structure determination of these different crystal forms present challenging combinations of pseudosymmetry, twinning and translational non­crystallographic symmetry.

## Abstract 2

An unusual case of protein–substrate complexes that were obtained under similar conditions but contain different packing arrangements is reported. The crystals exhibit a combination of various crystal imperfections (pseudosymmetry, twinning and translational non­crystallographic symmetry), masking the true crystal symmetry and challenging data processing and structure determination.

## 1. Introduction

Macromolecular crystals often suffer from imperfections that cause difficulties in space-group determination, data processing and refinement. Twinning is one of the most often encountered crystal defects. Different types of twinning are known. Epitaxial or nonmerohedral twinning is present when the lattices of the twin domains overlap in fewer than three dimensions, therefore making the diffraction patterns look abnormal. This kind of twinning can be detected by visual inspection of the diffraction images, and in some cases the diffraction spots belonging to the individual domains can be identified and processed separately (Liang *et al.*, 1996 ▸; Lietzke *et al.*, 1996 ▸). In contrast, merohedral twinning is characterized by a complete overlap of real-space lattices from the twin domains, resulting in superposition of the reciprocal lattice, and hence appearing normal (Yeates, 1997 ▸).

Merohedral twinning is detected by intensity distribution analyses, which will deviate from theoretical Wilson statistics due to the averaging of independent lattices that reduces the variation in intensity distributions (Wilson, 1949 ▸; Chandra *et al.*, 1999 ▸; Stanley, 1972 ▸). In merohedral twinning the holohedry belongs to a higher point group than the symmetry of the Laue class and therefore coset decomposition of the holohedry with regard to the Laue class is a method to determine the possible twin laws (Flack, 1987 ▸). These twin laws can be used to ‘detwin’ the crystal and calculate the true intensities in cases of twin fractions much smaller than 0.5 or to refine twinned crystal structures with use of the twin law in cases of perfect twinning (Yeates, 1997 ▸).

Merohedral twinning is only present in point groups belonging to crystal systems containing several Laue classes, such as point groups 3, 4, 6, 23 and 32 (hexagonal setting) (Yeates, 1997 ▸). Therefore, for point group 2 merohedral twinning is generally not possible, but there are exceptions. For example, in the fortuitous case of β ≃ 90°, an ortho­rhombic unit cell is mimicked and twinning becomes possible (Larsen *et al.*, 2002 ▸). Here, the holohedry exhibits *mmm* point-group symmetry, whereas the crystal structure has point group 2, which can be caused by two possible equivalent twin laws along **a** (*h*, −*k*, −*l*) or **c** (−*h*, −*k*, *l*). This kind of twinning is called pseudomerohedral twinning, with the orthorhombic and monoclinic point groups belonging to two different crystal systems (Parsons, 2003 ▸). In contrast to merohedral twinning, the lattices of the different twin domains overlap only approximately in three dimensions and therefore the spots in the diffraction pattern will not superpose completely (Yeates, 1997 ▸), often appearing as streaky reflections.

Pseudosymmetry is another phenomenon that may mask the true crystal symmetry. It is often observed when non­crystallographic symmetry (NCS) operators lie close to crystallographic operators and, as in the case of twinning, the holohedry has a higher point group than the crystal. Problems related to high *R* factors during refinement are a typical hallmark of pseudosymmetry (Zwart *et al.*, 2008 ▸).

A third common crystal phenomenon that impacts data processing and structure refinement is translational NCS (tNCS). Here, the NCS-related molecules are only related by a translation, while their orientation stays almost the same. This leads to a modulation of the diffraction pattern by the existence of systematic weak and strong spots arising from the fact that the related molecules contribute similar structure-factor amplitudes but different phases (Read *et al.*, 2013 ▸). Trans­lational NCS can be detected in the Patterson map by the presence of non-origin peaks with a height of at least 20% of the origin peak. The smaller the difference in orientation between the tNCS-related molecules, the more significant the effect of tNCS will be on data processing, phasing and refinement.

In this report, we describe three different crystal forms of the multihydrophobic amino-acid transporter (MhsT) with complications of pseudosymmetry, different degrees of pseudo­merohedral twinning and translational NCS. MhsT is an amino-acid transporter from *Bacillus halodurans* that transports a variety of hydrophobic l-amino acids. It is an orthologue of the mammalian neurotransmitter:sodium symporters and amino-acid transporters of the SLC6 family. MhsT substrates range from the bulky, aromatic substrates tryptophan, tyrosine and phenylalanine to the smaller, branched aliphatic amino acids valine, leucine and isoleucine (Quick & Javitch, 2007 ▸). The initial aim of the study was to understand the determinants of substrate specificity of MhsT through co-crystallization with all substrates. Six different structures were determined and, together with the previously published MhsT–Trp complex (Malinauskaite *et al.*, 2014 ▸), a substrate-recognition mechanism involving the unwound part of TM6 was elucidated (Focht *et al.*, 2021 ▸). Somewhat surprisingly, however, we observed that different crystal forms emerged but displayed related intermolecular crystal-packing arrangements. MhsT in complex with 4-fluorophenylalanine (4-F-Phe), Tyr, Phe and the previously determined Trp crystallize in space group *P*2 with the long axis along **c**. In the case of the smaller ligands (Val and Leu) a slight change in packing occurs and the space group changes to *P*2_1_, with new unit-cell parameters: 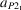 = *a*_*P*2_, 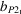 = 2*c*_*P*2_, 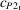 = *b*_*P*2_ and β ≃ 90°. The unit cell in the *P*2_1_ crystal form is approximately twice the volume of that in the *P*2 form and accommodates two MhsT complexes instead of one in the asymmetric unit, related by rotational NCS. The *P*2 form was never observed for the smaller aliphatic substrates, but the *P*2_1_ crystal form, on the other hand, was also observed for the aromatic substrates, although higher quality data sets were obtained in *P*2.

Another crystal-packing variation occurred in the case of the MhsT–Ile complex, which crystallized in a different *P*2_1_ crystal form, now with unit-cell parameters 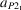 = *a*_*P*2_, 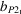 = 2*b*_*P*2_ and 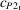 = *c*_*P*2_. Again, the unit cell is twice as large as for the *P*2 form, with the asymmetric unit containing two MhsT molecules; however, they are now related by translational NCS. Table 1 ▸ presents an overview of the different complexes, with a description of the space group, unit-cell parameters and data statistics.

While data processing and refinement were straightforward in the case of structures determined in space group *P*2, the *P*2_1_ cases turned out to be more challenging, especially because of the presence of pseudosymmetry and twinning in the Val- and Leu-bound complexes and of translational NCS in the case of MhsT–Ile, obscuring space-group assignment and refinement.

## 2. Methods

### 2.1. Protein expression, purification and crystallization

The expression, purification and crystallization of MhsT in complex with its different substrates have been described previously (Focht *et al.*, 2021 ▸) based on earlier studies (Malinauskaite *et al.*, 2014 ▸; Quick & Javitch, 2007 ▸).

### 2.2. Crystal morphology and data collection

**Figure 1:**
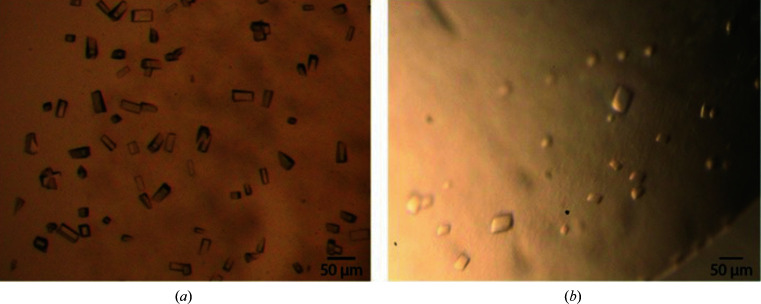
Crystals of MhsT. (*a*) *P*2_1_ crystal form of MhsT–Val exhibiting *P*222_1_ pseudosymmetry and negligible twinning and (*b*) a similar *P*2_1_ crystal form of MhsT–Leu with twin fraction 0.43. (*c*) MhsT–Ile crystallizes in a different *P*2_1_ form with translational noncrystallographic symmetry

Crystals of MhsT in complex with aromatic substrates (Phe, 4-F-Phe or Tyr), Val or Leu were small, three-dimensional rod-like crystals (Fig. 1 ▸ *a*) with lengths ranging from 30 to 50 µm, similar to MhsT–Trp crystals (Malinauskaite *et al.*, 2014 ▸). Data collection was performed on beamlines I24 and I04 at Diamond Light Source (DLS).

In contrast, the crystals of MhsT–Ile were flat plates with dimensions of up to 70 µm and thicknesses of about 5–10 µm (Fig. 1 ▸ *b*). The data sets were collected on beamline PXI at the Swiss Light Source (SLS). The MhsT–Ile crystals were very sensitive to radiation damage and complete data sets could not be obtained from single crystals.

## 3. Two data sets with pseudosymmetry and pseudomerohedral twinning

### 3.1. Data processing of MhsT–Val and MhsT–Leu data sets

The MhsT–Val and MhsT–Leu data sets were initially processed using the *XDS* package (Kabsch, 2010 ▸) as well as *POINTLESS* and *AIMLESS* from the *CCP*4 package (Winn *et al.*, 2011 ▸) in the orthorhombic space group *P*222_1_ with systematic absences along **c**. The processing resulted in an overall *R*_meas_ of 0.233 in the case of MhsT–Val and 0.133 in the case of MhsT–Leu, indicating seemingly acceptable merging statistics and space-group assignment. Initial phases were obtained by the use of molecular replacement in *Phenix Phaser-MR* with Mhst–Trp (PDB entry 4us3; Malinauskaite *et al.*, 2014 ▸) without TM5 and ligands as a search model, which identified one molecule in the asymmetric unit (Matthews coefficient of 2.57 Å^3^ Da^−1^ and solvent content of 52.1% for MhsT–Val and Matthews coefficient of 2.55 Å^3^ Da^−1^ and solvent content of 51.9% for MhsT–Leu; Matthews, 1968 ▸). Molecular replacement gave clear solutions in both cases (PAK = 0, LLG = 822 and TFZ = 18.2 for MhsT–Val and PAK = 0, LLG = 1262 and TFZ = 19.1 for MhsT–Leu), However, real- and reciprocal-space refinement stalled in both cases at an *R*_work_ and *R*_free_ of about 0.38 and 0.43, respectively.

These *R*-factor statistics indicated that the structure did not explain the diffraction data well, and that the assignment of *P*222_1_ space-group symmetry was potentially incorrect. The presence of pseudosymmetry and/or twinning in the data sets was suspected to make the diffraction pattern resemble an orthorhombic space group due to a fortuitous value of β ≃ 90°. Therefore, the three monoclinic *Translationengleiche* sub­groups of *P*222_1_ were explored to investigate which twofold or screw operator present in the orthorhombic space group remained valid (if any) as a crystallographic operator in a monoclinic space group.

In the case of MhsT–Val, the merging *R* factors for the two different settings of space group *P*2 were markedly increased (Table 2 ▸), suggesting that they also were not valid space groups for this data set, and indeed they also resulted in high model *R* factors in refinement. However, processing in space group *P*2_1_ with the long axis (the *c* axis in *P*222_1_) now along **b** merged with proper statistics and an overall *R*_meas_ of 0.138. Molecular replacement located two molecules in the asymmetric unit (Matthews coefficient of 2.57 Å^3^ Da^−1^, solvent content of 52.1%), yielding a single solution with PAK = 0, LLG = 5863.578 and TFZ = 53.9. Model refinement proceeded smoothly and converged with an *R*_work_ and *R*_free_ of 0.207 and 0.237, respectively, with high-quality electron-density maps for unmodeled parts.

Similarly, parallel runs of molecular replacement and initial refinement were performed for MhsT–Leu, yielding initial *R*_work_ and *R*_free_ values of 0.26 and 0.31, respectively, for *P*2_1_, 0.35 and 0.42, respectively, for *P*2_*b*=44.26 Å_ and 0.31 and 0.37, respectively, for *P*2_*b*=50.17 Å_. These results clearly indicated that space group *P*2_1_ was again the correct assignment, similar to MhsT–Val. The complete processing and refinement statistics for the two data sets processed in *P*2_1_ can be seen in Table 3 ▸.

However, for MhsT–Leu the data-scaling statistics looked comparable in all three monoclinic assignments, *i.e.* with the two *P*2 space groups having only a slightly increased *R*_measure_ compared with *P*2_1_. This seemingly different behaviour of the MhsT–Val and MhsT–Leu data was investigated further and is explained below.

### 3.2. Pseudosymmetry of the MhsT–Val and MhsT–Leu crystals

**Figure 2:**
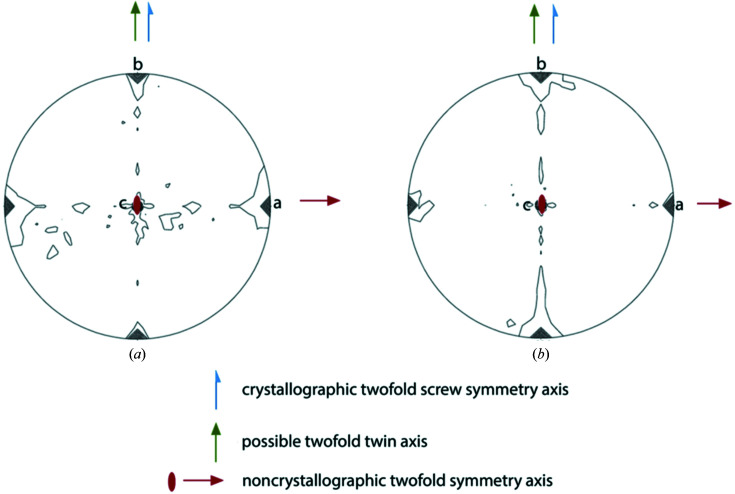
Self-rotation function of (*a*) MhsT–Val and (*b*) MhsT–Leu in the κ = 180° section. The low-resolution limit is 7 Å, the high-resolution limit is 3 Å and the radius of integration is 32 Å. The crystallographic twofold screw symmetry axis is present along **b**, the noncrystallographic twofold symmetry axis and twin axis are present along **a** and the third twofold axis is present along **c**.

The presence and orientation of crystallographic and non­crystallographic rotational and screw axes in the two data sets were further analysed by use of the Patterson self-rotation function (Rossmann & Blow, 1962 ▸). Fig. 2 ▸ presents a stereographic projection of the self-rotation function at κ = 180°, indicating 222 symmetry or 222 pseudosymmetry with the NCS axes lying close to the crystallographic axes, which are therefore also candidates as a possible twin axis.

The *P*222_1_ pseudosymmetry prompted us to investigate the NCS operators with ‘Find NCS operators’ in *Phenix*. The following operators were found for the two data sets. For MhsT–Leu, 

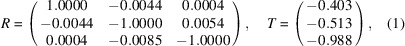

and for MhsT–Val,

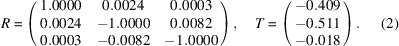

Hence, the NCS operator approximates a crystallographic twofold axis along **a**, which together with the crystallographic twofold axis along **b** generates a third twofold operator parallel to **c** and a *P*222_1_ pseudosymmetry. This operator is assumed to be crystallographic when processing is performed in the orthorhombic space group, but because the NCS operator diverges from the crystallographic operator the refinement in the orthorhombic space group stalls at high model *R* factors, as indicated above.

### 3.3. Pseudomerohedral twinning of MhsT–Leu

**Figure 3:**
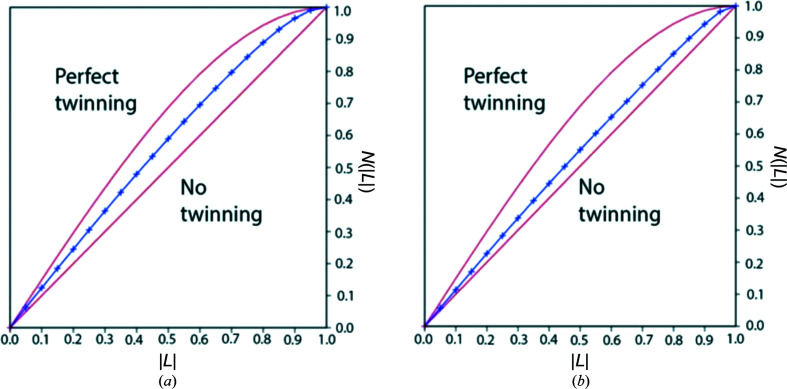
Graphs presenting statistics in the (*a*) MhsT–Leu and (*b*) MhsT–Val data sets.

Intensity analyses were performed on the monoclinic data sets in *phenix.xtriage* (Zwart *et al.*, 2005 ▸) as β approaches 90° for both MhsT–Val and MhsT–Leu, making pseudomero­hedral twinning possible. The Wilson ratio and secondary intensity moments (Stanley, 1972 ▸; Rees, 1980 ▸) of centric and acentric reflections hinted at twinning in both data sets (data not shown) with the *h*, −*k*, −*l* twin law. Additionally, a more robust local intensity difference analysis of the data sets was performed by use of The Merohedral Twinning Server (https://services.mbi.ucla.edu/Twinning/) applying the Padilla–Yeates algorithm (Padilla & Yeates, 2003 ▸). The presence of twinning is indicated in both data sets (Fig. 3 ▸), but with a markedly higher degree of twinning in the case of MhsT–Leu.

The presence of a twofold NCS operator along **a** can bias twinning detection to indicate a twin operator along **a**, because the analysis of the scaling statistics assumes that the related reflections are otherwise independent (Yeates & Rees, 1987 ▸; Lebedev *et al.*, 2006 ▸), although it is already clear from the NCS operator that they are not. At the same time, cases in which the NCS operator coincides with a crystal axis make twinning highly probable (Lebedev *et al.*, 2006 ▸). Generally, twin fractions can be determined in several ways (Britton, 1972 ▸; Rees, 1980 ▸; Fisher & Sweet, 1980 ▸; Murray-Rust, 1973 ▸; Yeates, 1997 ▸); however, many of these tests do not give an accurate estimate of the twin fraction in cases such as this. Therefore, a maximum-likelihood test that takes the NCS axis into consideration was used in *phenix.xtriage* (Liebschner *et al.*, 2019 ▸). The obtained twin fraction for MhsT–Val was 0.065, while it was 0.443 for MhsT–Leu. In comparison, the Britton test (Britton, 1972 ▸) gave twin fractions of 0.173 for MhsT–Val and 0.447 for MhsT–Leu.

A significant decrease (for example 3–10%) in model *R* factors is to be expected in cases with high degrees of twinning when comparing refinement with and without a twin law. Indeed, for the MhsT–Leu data set structure refinement with the *h*, −*k*, −*l* twin law resulted in a decrease in the refinement *R* factors from an *R*_work_ and *R*_free_ of 0.266 and 0.298, respectively, to 0.185 and 0.222. However, in the MhsT–Val complex with a low degree of twinning, *R*_work_ and *R*_free_ were 0.207 and 0.237, respectively, without the use of the twin law and 0.194 and 0.223, respectively, when the *h*, −*k*, −*l* twin law was used (a refined twin fraction of 0.06); *i.e.* there was an almost negligible difference. As refinement with the twin law also did not improve the electron-density maps for MhsT–Val, we refined the MhsT–Val structure without the use of the twin law.

The significant difference in the twin fractions observed between the two data sets explains the low merging factors of the MhsT–Leu data set in all three monoclinic subgroups of *P*222_1_. In the case of a data set belonging to *P*222_1_, proper scaling and low merging factors would be expected in its type I maximal non-isomorphous subgroups. Therefore, in the case of two monoclinic data sets containing twin fractions of ∼0 and ∼0.443 it could be expected that the data set with almost perfect twinning would scale better in all three subgroups, as the diffraction pattern approximates an orthorhombic setting more closely than the data set with the lower twin fraction. However, in the case where pseudosymmetry is present at the same time, as here, the merging statistics will also appear valid in the case of *P*222_1_, even though the twin fraction is small or non-existent (Parsons, 2003 ▸).

### 3.4. Crystal packing explains the pseudosymmetry of MhsT–Val and MhsT–Leu

**Figure 4:**
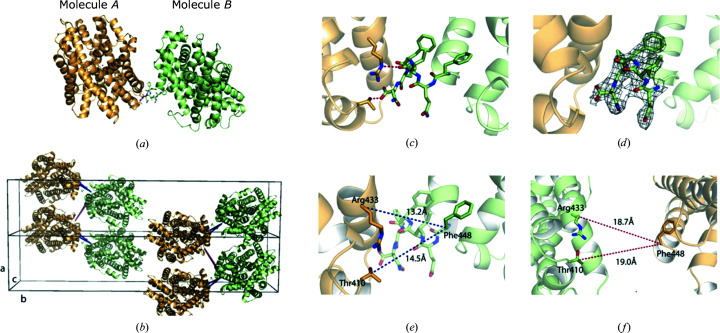
(*a*) Two molecules in the asymmetric unit of MhsT–Val, also representing MhsT–Leu. (*b*) Two unit cells are shown. Distances between molecules in the asymmetric unit are shown in blue and distances between molecules in different asymmetric units are shown in red. (*c*) Interactions between the C-­terminus in molecule *B* and two residues in molecule *A*. (*d*) 2*F*_o_ − *F*_c_ electron-density map of the additional residues in the C-terminus of molecule *B* contoured at 1 r.m.s.d. (*e*) Distances between molecules in the asymmetric unit. (*f*) Distances between molecules in different asymmetric units.

In order to visualize the pseudosymmetry in the crystal structure, a closer analysis of the crystal packing was made. The two molecules in the *P*2_1_ asymmetric unit, molecules *A* and *B* (Figs. 4 ▸ *a* and 4 ▸ *b*), are related by twofold rotational NCS, with a C^α^ r.m.s.d. of 0.232 Å. Smaller conformational deviations between the molecules are present in the loop regions 248–251 and 419–424. However, a main difference is that the C-terminal end of molecule *A* can only be traced to Phe448, whereas for molecule *B* the entire C-terminus ending at Asn453 is visible in the maps (Fig. 4 ▸ *d*), with this region being stabilized by local interactions with the neighbouring molecule *A* (Fig. 4 ▸ *c*). In the case of molecule *A*, however, the distance between the C-terminus and molecule *B* is larger and no interaction is observed (Fig. 4 ▸ *f*). In other words, molecules *A* and *B* are not identical, the symmetry operations superimposing them are imperfect and orthorhombic symmetry is not present.

The variations in crystal packing may be due to subtle ligand-induced conformational changes of the MhsT structure, and we also cannot exclude that the presence of amino-acid ligands at ∼0.5 m*M* concentration may affect the lipid–detergent phase diagram and therefore the crystallization conditions. As mentioned earlier, the aromatic substrate complexes can crystallize in both *P*2 and *P*2_1_ forms, and we therefore performed a systematic crystallization approach with controlled protein:lipid ratios for the MhsT–Trp complex. At protein:lipid ratios of 3:0.8(*w*:*w*) and 3:1.0(*w*:*w*) MhsT–Trp mainly crystallized in the *P*2_1_ form, whereas at a ratio of 3:2.25 it crystallized mainly in the *P*2 form. These ratios are dependent on both the protein and lipid batch, and cryoprotection procedures also seem to have an effect, but it seems clear that, for example, protein:lipid ratios affect crystal-packing preferences and highlight the importance of exploring and controlling these ratios in crystal screening and optimization.

## 4. Data set with pseudo-translation: MhsT-Ile

### 4.1. Data processing and pseudo-translation of MhsT–Ile

The data sets obtained from the MhsT–Ile crystals were processed with the *XDS* package (Kabsch, 2010 ▸) and the *CCP*4 suite of programs (Winn *et al.*, 2011 ▸) as described for the other complexes, although autoindexing failed in some cases. For most crystals a data set could not be collected due to low resolution or poor quality of the diffraction, with streaky or split reflections. The crystal form was again monoclinic, but it was unclear whether systematic absences were present along **b** because this direction had been poorly sampled by the data-collection runs before the onset of radiation damage and also due to the anisotropic diffraction properties of the thin plate crystals in the loop. Furthermore, the crystals were generally not isomorphous, but two fairly isomorphous data sets were identified and merged, and despite a low completeness of ∼80% the data were of sufficient quality that we could distinguish space-group assignments and perform structure determination and limited refinement. Important structural features of the complex, especially inside the binding pocket, could be obtained and compared with the other substrate complexes (Focht *et al.*, 2021 ▸).

Importantly, analysis of the data set in *phenix.xtriage* revealed a non-origin peak with a size of 59.6% of the origin peak in the Patterson map at fractional coordinates (0.337, 0.5, −0.312), indicating translational NCS (tNCS).

**Figure 5:**
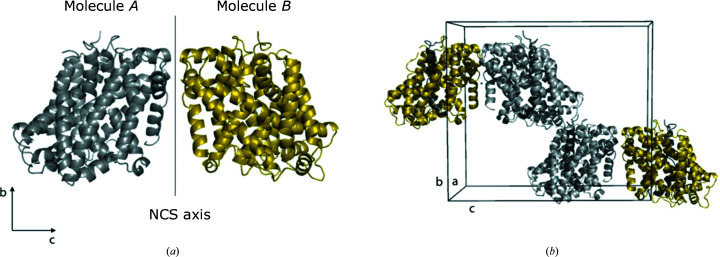
Visualization of the translational noncrystallographic symmetry of MhsT–Ile. (*a*) Two molecules, *A* and *B*, related by NCS. The NCS axis is parallel to **b**. (*b*) Molecule *A*_1_ has almost the same orientation as molecule *B*_2_ and these two molecules are only related by translation. TM1 is coloured in dark grey to more easily visualize the orientation of the molecules.

As we were unable to distinguish *P*2 and *P*2_1_ in scaling without data along **b**, molecular replacement was performed in both space groups. Molecular replacement was performed in *Phenix Phaser-MR* using MhsT–Trp (PDB entry 4us3 without TM5 and ligands) as a search model, proposing a model in space group *P*2_1_ with two molecules in the asymmetric unit (Matthews coefficient 2.52 Å^3^ Da^−1^, solvent content 51.2%). However, the model exhibited negative LLG values and *R* factors close to 50–60%. Additionally, the model could not explain the presence of the large non-origin peak in the Patterson map. When molecular replacement was extended to *P*1, searching for four molecules in the unit cell, different relationships between the molecules were revealed. Here, as expected, two pairs of molecules could be distinguished; however, surprisingly, they were related by a twofold NCS axis almost parallel to **b**. Discovering the possible relationships in the asymmetric unit and guided by the non-origin peak in the Patterson map, models of molecules were created by the use of ‘Apply NCS operators’ in *Phenix* in space group *P*2_1_, with two molecules related by a NCS twofold axis parallel to **b** with the translational matrix derived from the non-origin peak in the Patterson map, as only this relationship would explain the coordinates of the peak. A small deviation (only 0.23°) in the orientation of the molecules related by tNCS (Fig. 5 ▸ *b*) to­gether with the streaky reflections explained the problems with indexing and data processing of the data sets (Read *et al.*, 2013 ▸).

**Figure 6:**
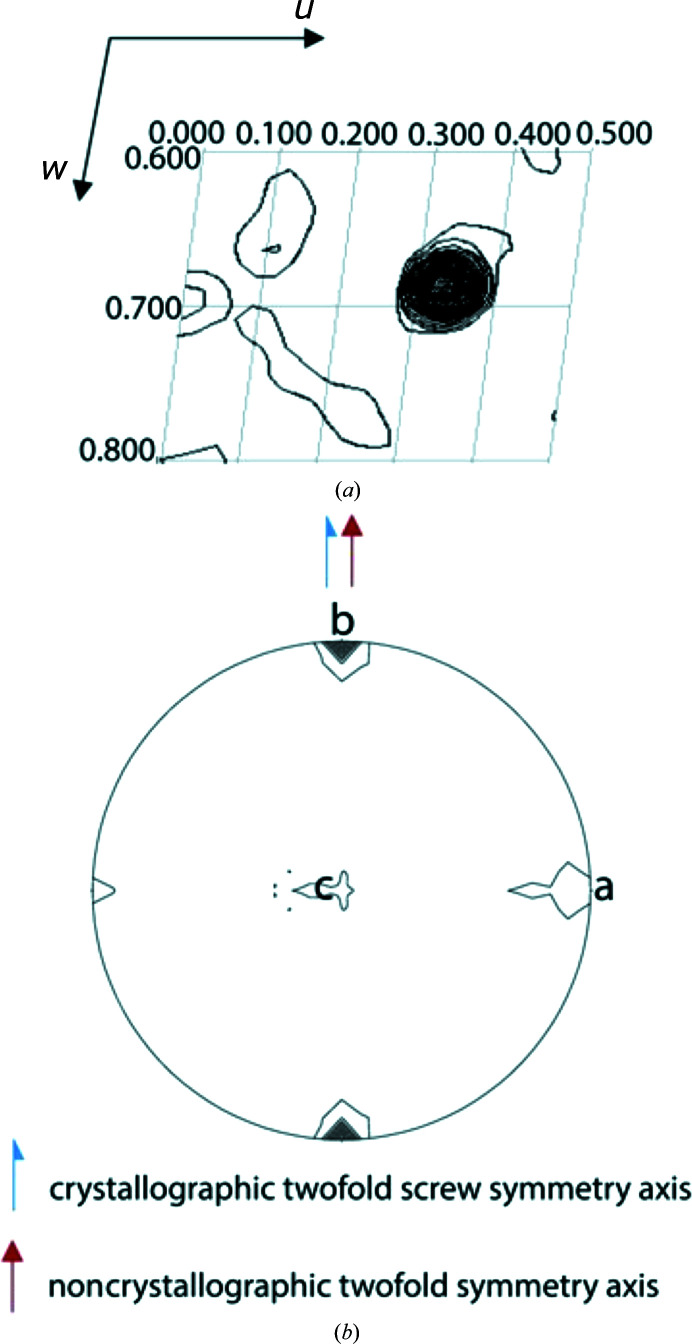
(*a*) Harker section at *v* = 0.5 of the Patterson map for MhsT–Ile visualizing the non-origin peak with a size of 59.6% of the origin peak. The map is drawn with a minimum contour level at 1.0σ with 1.5σ increments. (*b*) Self-rotation function of MhsT–Ile. The crystallographic twofold screw axis as well as the NCS axis are both present along **b**.

Fig. 6 ▸(*a*) presents the Harker section at *v* = 0.5, whereas Fig. 6 ▸(*b*) shows the stereographic projection of the self-rotation function at κ = 180° with the crystallographic twofold screw axis and the noncrystallographic twofold axis both along **b**.

This solution not only explained the non-origin peak in the Patterson map, but also caused an immediate decrease in the *R* factors to an *R*_work_ and *R*_free_ of 0.313 and 0.347, respectively, in the initial round of refinement. The model was further refined in *phenix.refine*, ending at a final *R*_work_ and *R*_free_ of 0.277 and 0.305, respectively, which was deemed to be acceptable considering the presence of tNCS, the low completeness of the data and the overall lower resolution and quality of the data set. However, combined with an accurate and overall identical model from other high-resolution structures, a meaningful analysis could be obtained (Focht *et al.*, 2021 ▸). Processing and refinement statistics are summarized in Table 3 ▸.

## 5. Discussion

We present a remarkable case of almost identical complexes of the amino-acid transporter MhsT crystallized with different amino-acid substrates that however exhibit a range of crystallographic phenomena, including variable space-group symmetries, pseudosymmetry, different degrees of pseudomerohedral twinning and translational NCS. These variations challenged space-group determination, data processing and model refinement. It is worth noting that in the two first cases (MhsT–Val and MhsT–Leu) excellent electron-density maps were obtained in the pseudo-orthorhombic setting that, if combined with the unreasonable presumptions of membrane-protein crystal structures being allowed to pass lower quality thresholds, could lead to incorrect space-group assignments and structures. In the case of twinning, at a low/absent twin fraction (the MhsT–Val complex) it remains possible to discern the correct monoclinic subgroup over orthorhombic pseudosymmetry through the careful comparison of merging statistics for the individual monoclinic subgroups. However, this becomes difficult when almost perfect twinning (the MhsT–Leu complex) occurs and the merging statistics become essentially indistinguishable for all three monoclinic sub­groups. In this case only model refinement allowed us to distinguish the correct monoclinic space-group assignment, even without twin refinement.

In the case of MhsT–Val and MhsT–Leu, the cause of pseudosymmetry can directly be identified in the crystal packing as a significant difference in local interactions around the C-terminus, making two molecules, *A* and *B*, non-identical. The twin operation scrambles the distinction of *A*–*B* and *B*–*A* pseudosymmetry pairs. We observe that variations in the protein:lipid ratios can also affect the resulting crystal-packing symmetry of the MhsT–Trp complex.

Different cases of proteins determined in monoclinic *P*2_1_ forms with pseudomerohedral twinning have previously been described. Larsen *et al.* (2002 ▸), Barends & Dijkstra (2003 ▸) and Golinelli-Pimpaneau (2005 ▸) described cases in which a primitive orthorhombic symmetry is mimicked, similar to the case of MhsT. Other kinds of pseudomerohedral twinning in a monoclinic space group that impose an apparent higher symmetry can also be present, for example when *c* cos(β) = −*a*/2 (Declercq & Evrard, 2001 ▸; Rudolph *et al.*, 2004 ▸) or when *a* ≃ *c* (Ban *et al.*, 1999 ▸; Yang *et al.*, 2000 ▸).

An analysis of entries in the Protein Data Bank (PDB) reveals that the described types of pseudosymmetry and pseudomerohedral twinning can potentially occur quite often. Careful intensity and model-refinement analyses are warranted in such cases. By considering structures determined by X-ray crystallography with experimental data available (analysis performed on 20 April 2022), of a total of 165 026 structures 27 568 (16.7%) were determined in the monoclinic space group *P*2_1_. After *P*2_1_2_1_2_1_ it is the second most populated space group in the PDB, and is followed by *C*2. 1901 of these *P*2_1_ structures have a β angle between 89° and 91° (6.9%, excluding 128 entries that contain only one molecule in the asymmetric unit), where pseudomerohedral twinning must be assumed as a potential descriptor. Similarly, cases with model refinement stalling at suspiciously high *R* factors obviously warrant careful consideration of incorrect space-group assignment, pseudosymmetry and potentially twinning, where an incorrect *P*1 assignment should also be avoided.

## Figures and Tables

**Table 1:** Space groups and unit-cell parameters of the various MhsT complexes

Data set	MhsT + Trp†	MhsT + 4-F-Phe	MhsT + Tyr	MhsT + Phe	MhsT + Val	MhsT + Leu	MhsT + Ile
Space group	*P*2	*P*2	*P*2	*P*2	*P*2_1_	*P*2_1_	*P*2_1_
Resolution (Å)	2.1	2.26	2.3	2.25	2.6	2.35	3.2
*a*, *b*, *c* (Å)	44.3, 49.9, 110.1	44.2, 49.9, 109.7	44.1, 49.9, 110.3	44.4, 49.9, 110.1	44.1, 216.1, 50.4	44.2, 215.6, 50.2	44.0, 97.3, 110.3
α, β, γ (°)	90.0, 96.8, 90.0	90.0, 96,1, 90.0	90.0, 96,8, 90.0	90.0, 96.8, 90.0	90.0, 90.0, 90.0	90.0, 90.1, 90.0	90.0, 90.1, 90.0
No. of molecules in asymmetric unit	1	1	1	1	2	2	2
Twin law	N/A	N/A	N/A	N/A	*h*, −*k*, −*l*	*h*, −*k*, −*l*	N/A
Twin fraction	N/A	N/A	N/A	N/A	0.065	0.443	N/A

† Data from Malinauskaite *et al.* (2014 ▸).

**Table 2:** Processing of MhsT–Val and MhsT–Leu in different space groups

	MhsT–Val			MhsT–Leu		
Space group	*a*, *b*, *c* (Å)	α, β, γ (°)	*R*_meas_	*a*, *b*, *c* (Å)	α, β, γ (°)	*R*_meas_
*P*1	44.11, 50.31, 215.79	89.99, 89.91, 90.02	0.109 (0.708)	44.25, 50.21, 215.70	89.85, 90.03, 90.05	0.117 (0.896)
*P*222_1_	44.17, 50.37, 216.03	90, 90, 90	0.233 (1.268)	44.26, 50.21, 215.71	90, 90, 90	0.133 (1.027)
*P*2_*b*=50.31 Å_	44.12, 50.31, 215.81	90, 90.09, 90	0.216 (1.001)	44.23, 50.17, 215.55	90, 90.02, 90	0.131 (0.893)
*P*2_*b*=44.17 Å_	50.37, 44.17, 216.07	90, 90.01, 90	0.196 (0.965)	50.21, 44.26, 215.71	90, 90.14, 90	0.124 (0.967)
*P*2_1_	44.12, 216.07, 50.37	90, 90.02, 90	0.138 (1.105)	44.23, 215.56, 50.17	90, 90.05, 90	0.117 (0.849)

**Table 3:** Processing and refinement statistics for MhsT–Val, MhsT–Leu and MhsT–Ile Values in parentheses are for the highest resolution shell.

	MhsT–Val	MhsT–Leu	MhsT–Ile
Data collection			
Beamline	I02, DLS	I04, DLS	PXI, SLS
Space group	*P*2_1_	*P*2_1_	*P*2_1_
*a*, *b*, *c* (Å)	44.1, 216.1, 50.4	44.2, 215.6, 50.2	44.0, 97.3, 110.9
α, β, γ (°)	90, 90.02, 90	90, 90.05, 90	90, 96.14, 90
Resolution range (Å)	49–2.60 (2.72–2.60)	45–2.35 (2.43–2.35)	43.7–3.10 (3.31–3.10)
No. of reflections (total/unique)	97704/28703	114897/38300	37578/13889
Wilson *B* factor (Å^2^)	49.3	37.4	63.0
Completeness (%)	99.5 (99.7)	98.5 (98.5)	81.6 (83.8)
*R*_merge_	0.117 (0.950)	0.096 (0.679)	0.195 (0.815)
*R*_p.i.m._	0.075 (0.607)	0.066 (0.502)	0.124 (0.532)
Mean *I*/σ(*I*)	8.0 (1.2)	8.1 (1.4)	3.6 (1.2)
Multiplicity	3.4 (3.4)	3.0 (2.6)	2.7 (2.5)
CC_1/2_	0.994 (0.489)	0.996 (0.511)	0.989 (0.636)
Twin fraction derived	0.065	0.443	N/A
Refinement			
Twin law†	Not used	*h*, −*k*, −*l*	N/A
*R*_work_/*R*_free_ ‡	0.207/0.237	0.185/0.222	0.277/0.305
No. of molecules in asymmetric unit	2	2	2
No. of atoms			
Protein	6674	6678	6630
Ligand	16	18	18
Ions	4	4	4
Detergent/lipid	357	176	47
Water	73	46	19
Average *B* factor (Å^2^)			
Protein	56.9	41.4	56.7
Ligand	48.8	34.1	52.6
Ions	49.6	33.3	52.8
Detergent/lipid	68.4	51.7	58.2
Water	55.4	37.9	52.2
R.m.s.d.			
Bond lengths (Å)	0.003	0.002	0.002
Bond angles (°)	0.595	0.505	0.578
Ramachandran statistics (%)			
Favourable	96.4	96.9	95.7
Outliers	0	0	0.23

† MhsT–Leu was refined against the *h*, −*k*, −*l* twin law, whereas MhsT–Val was refined without the twin law as it did not have any significant influence on the *R* factors during refinement.

‡ 5% of the data set was chosen for the *R*_free_ sets; additionally, in the cases with two molecules in the asymmetric unit the *R*_free_ flag was assigned in thin resolution shells.
